# Constraints on Development of Wind Energy in Poland due to Environmental Objectives. Is There Space in Poland for Wind Farm Siting?

**DOI:** 10.1007/s00267-016-0788-x

**Published:** 2016-11-03

**Authors:** Małgorzata Hajto, Zdzisław Cichocki, Małgorzata Bidłasik, Jan Borzyszkowski, Agnieszka Kuśmierz

**Affiliations:** 1 Institute of Environmental Protection, National Research Institute in Warsaw, ul. Krucza 5/11D, Warsaw, 00-458 Poland; 2 Institute of Environmental Protection, National Research Institute, Branch in Wroclaw, ul. Wybrzeże Wyspiańskiego 39e, Wroclaw, 50-370 Poland

**Keywords:** Landscape, Spatial planning, Environmental impact assessment, Poland, Wind farm

## Abstract

The objective of the study was to evaluate spatial effects of adopting environmental criteria for wind farm siting, i.e., the criteria related to the settlement system and those with regards to landscape values. The set of criteria was elaborated on the basis of literature and experience-based knowledge. Some of the criteria selected are legally binding. The analyses were carried out with the use of GIS tools. Settlement areas with 1000 and 2000 m wide buffer zones, and the areas with the highest landscape values, were assumed as particularly sensitive receptors to wind farm impacts. The results show significant constraints on wind farm siting in Poland. Although the constraints are regionally diversified, they concern 93.9 % of the total country area (1000 m buffer zone) or 99.1 % (2000 m buffer zone). Presumably even greater constraints would be revealed by an additional detailed analysis at a local level. The constraints on wind farm siting in Poland cannot be decreased, because of both social attitudes and demand for appropriate environmental standards, which should be taken into account in spatial and energy policies at all decision making level.

## Introduction

National energy policies, must be implemented at a local level, e.g., through wind farm siting. At a local level, it is necessary to determine a balanced relationship between environmental protection and investment needs (Hull 1995). Experiences in different European countries provide evidence to demonstrate that the supply of available space represents a serious constraint on wind energy policy implementation (Cowell 2010). Problems related to wind farm siting also concern Poland.

**Fig. 1:**
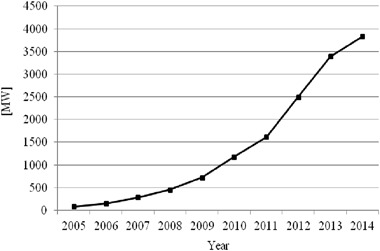
Wind power capacity [MW] in Poland. *Source*: Energy Regulatory Office of Poland 2014

**Fig. 2:**
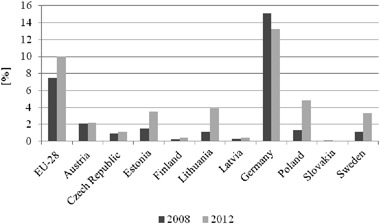
Share of wind-generated power in the energy produced from renewable energy sources in selected EU countries [%]. *Source*: Central Statistical Office of Poland 2014

Poland has been experiencing gradual development of wind energy as demonstrated by an increase in the total installed capacity (Fig. 1) and a growing share of the wind-generated power in the total energy produced from renewable energy sources (RES) (Fig. 2). From 2008 to 2012, wind energy share increased 3.5 times, and compared with other countries in the European Union this growth rate was the highest (Fig. 2).

Poland’s wind conditions are relatively favorable for wind energy development, however, they are regionally diversified (Lorenc 2005; Michalczuk 2011; Sliz-Szkliniarz and Vogt 2011). Northern regions of Poland and those in central Poland are distinctly most favorable for generation of wind energy. In terms of the number of installations and their power capacity, the current distribution pattern of wind farms reflects wind conditions in the country (Table 1).

**Table 1:** Number of installations and wind power capacity in Poland

Regions (Voivodships)	Number of installations	Capacity [MW]
Poland	931	3833.830
Dolnośląskie	10	162.365
Kujawsko-pomorskie	245	365.399
Lubelskie	6	3.650
Lubuskie	8	63.000
Łódzkie	188	386.180
Małopolskie	12	3.469
Mazowieckie	79	241.136
Opolskie	9	103.649
Podkarpackie	25	84.410
Podlaskie	24	151.400
Pomorskie	40	424.110
Śląskie	23	21.175
Świętokrzyskie	18	9.626
Warmińsko-mazurskie	31	271.575
Wielkopolskie	152	466.489
Zachodniopomorskie	61	1076.197

*Source:* Energy Regulatory Office of Poland 2014

The development of energy production based on RES is one of the priority objectives listed in the Energy Policy of Poland until 2030 (2009). In addition to different tools for policy implementation, the document includes e.g., “hierarchy-based spatial planning ensuring the implementation of energy policy priorities” (Energy Policy of Poland until 2030 2009). This means that the spatial planning system should involve the implementation of energy policy at all the governance levels: national (country), regional (Voivodship) and local (municipal). Hence, the regional and local authorities are expected to actively implement the energy policy. Among others, regional and local actions should comprise preparing strategies for development of energy production, including wind power. In Poland, just as in Sweden (Ek et al. 2013) and the United States (Huber et al. 2010), local governments have a good deal of authority over spatial planning, and the decisions on wind farm siting are taken at a local level. Recognizing economic benefits (local taxes) to be gained from wind farms siting, some local governments try to attract investors with specific spatial policy. For financial reasons, many local governments specify the areas available for wind farms siting in the documents on spatial planning (acts of local law). This approach often differs from expectations of local communities, and public opposition against wind farms is increasing.

By the 2100s, developers of wind farms in Poland could expect a positive attitude on the part of local governments and communities. However, presently, many social conflicts have been observed and the process of development of wind farms has become “an uphill battle” (Wolsink 2007). It seems that in wind farm siting, the decisive factor is economic gain since a wind farm brings financial benefits to local communities. Agterbosch et al. (2009) pointed out that local communities started to oppose a wind farm project, when recognized that the positive decision would serve external economic interests, while ignoring the risks for the residents or local landscapes and nature. The cited authors called this “an informal and top-down decision-making strategy”. It also seems that the system of environmental assessments (strategic environmental assessments—SEAs and environmental impact assessments—EIAs) does not fulfill own objectives. SEAs and EIAs are the tools that support stakeholder negotiations on acceptable wind farm locations, but they are not credible to local communities (Cowell 2010; Eltham et al. 2008; Hull 1995; Smart et al. 2014). Also, they are not sufficiently used at the stage of investment planning (Cowell 2010; Eltham et al. 2008; Hull 1995; Van der Horst and Toke 2010). Consequently, as the number of built turbines continues to grow, more and more opponents emerge.

The factors related to opposition to wind power plants and their relationships between have been analyzed in a number of studies. There have been pointed out the aspects such as: landscape features (Cowell 2010; Wolsink 2007), protesters’ attitudes (Baxter et al. 2013; Bidwell 2013; Groth and Vogt 2014), the procedures on planning and decision-making (Eltham et al. 2008; Smart et al. 2014; Van der Horst and Toke 2010; Wolsink 2007) and features of democracy (Breukers and Wolsink 2007; Van der Horst and Toke 2010). Pepermans and Loots (2013) reviewed the factors related to the protests against wind farms, i.e.: fears concerning impacts of wind farms on human health and the landscape, attachment to the land, procedures and access to the judiciary, social cohesion, social involvement as well as relationships between the planning system and ownership. As stated by Agterbosch and Breukers: “wind turbines are the source of multiple conflicts over interests and meanings” (in: Pepermans and Loots 2013), however, Sturge et al. (2014) and Van der Horst and Toke (2010) suggested that the most important issue was keeping a certain minimum distance between wind turbines and residential areas.

In the present study we reflect on whether after fulfilling stakeholder expectations concerning the distance between a wind farm and residential area, there still will be space available for establishing wind farms. In Poland, no assessment as such has been carried out so far. In other European countries and the United States, several analyses have been performed to indicate the strategic areas for wind energy development, but in comparison with Poland, there are completely different settlement structures. At present, in Poland there is a discussion underway about the crisis in spatial planning, which is mainly manifested by dispersal of settlements. This situation is not favorable for the siting of many different investment projects with possible adverse impacts on humans, including wind farms.

The overall objective of the present study was to evaluate spatial effects of adopting environmental criteria for wind farm siting, i.e., the criteria related to the settlement system and taking into account landscape conservation. In particular, the study attempted to:

- compare the regions of Poland in terms of constraints on wind farm siting, with the use of settlement-related and landscape-related criteria;
- reveal importance of settlement-related and landscape-related criteria, depending on the region evaluated.

The article presents the conceptual framework which we followed in choosing the criteria for wind farm siting. The concept of environmental sensitivity to wind farm impacts was of key importance. We applied the selected criteria in spatial GIS analyses, using the available databases. We identified the distribution of constraints on wind farm siting in individual regions of the country. The study results include statistical data and maps showing the described constraints in Poland and its regions. An important issue which emerged in the discussion is the level of analyses, as it affects the results: the constraints observed at a regional level would be even greater if the analysis concerned a local level.

## Conceptual Framework

### Theoretical Grounds

This section presents the approach to the selection of criteria limiting the spatial possibilities of wind farms. We refer to the theoretical grounds for environmental impact assessments as well as to the concept of the sensitivity of the environment to wind farm impacts.

Each part of space has its specific carrying capacity with respect to development forms which fill a given area. When the capacity is exceeded by a given form of use, the space is degraded as a whole, and thus its ability to meet human needs is reduced (Kostrowicki 1992). The carrying capacity depends on both the development forms, which are already present in a given space, and a new form which is to be introduced. All existing forms are considered to be the receptors of new element impacts (Pavlickova and Vyskupova 2015; Toro et al. 2012). Consequently, space capacity of the space is determined by the existing receptors, which are sensitive to an adverse impact of a new development form.

Sensitivity is a feature of these environment elements which risk a loss of their values under changed conditions (Bradley and Smith 2004; Pavlickova and Vyskupova 2015; Toro et al. 2012). A new development form may change environmental conditions for the worse, nevertheless, environmental impact assessments aim at preserving the values. The goals of the SEAs and EIAs are to determine such conditions for the new development, under which the existing receptor is able to function without risking a loss of its values (Pavlickova and Vyskupova 2015; Toro et al. 2012).

The choice of the sensitive receptors provided the basis for laying down the criteria for further analyses. There are the following types of the receptors particularly sensitive to wind farm impacts:

1. humans—especially due to wind farm visual impacts and sound emissions, (both perceived as annoying) (Cowell 2010; Möller 2010; Pedersen 2011; Pawlaczyk-Łuszczyńska et al. 2014; Wolsink 2010);
2. animals (in particular birds and bats)—because of possible collision with rotating blades, wind turbine sound emissions, as well as due to limitation of animal living space (e.g., breeding, wintering, resting, and feeding grounds or migration routes) (Kunz et al. 2007; Pearce-Higgins et al. 2012; Rodrigues et al. 2015; Schuster et al. 2015);
3. landscape—because of the a change in the landscape structure and wind farm adverse impacts on landscape aesthetic values (Baxter et al. 2013; Kokologosa et al. 2014; Wolsink 2010).

**Fig. 3:**
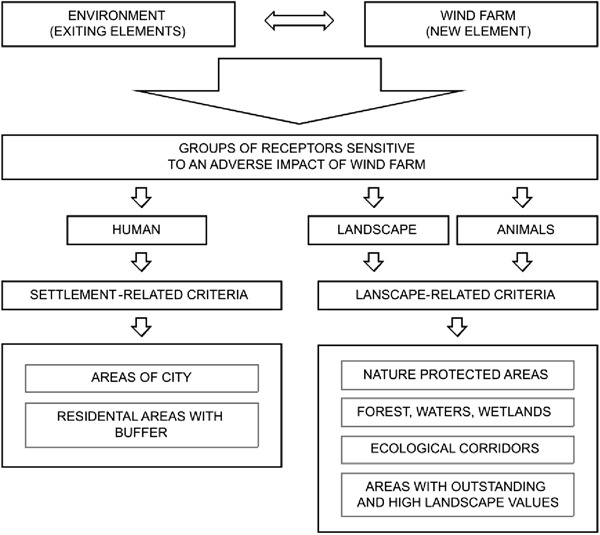
The conceptual framework adopted in the study

For the purpose of the present study, the receptors were assigned to 2 groups: (1) the settlement-related criteria and (2) the landscape-related criteria. The adopted criteria (Fig. 3) either exclude or limit the possibility of wind farm siting and are further described in the section below.

### Selection of Criteria

The adopted conceptual framework served as the basis for the selection of the criteria with reference to environment sensitivity to wind farm adverse impacts. The analyses of the constraints on wind farm development in the country were carried out at a regional level. Hence, the occurrence of sensitive receptors was identified in the specific zones (natural or functional and administrative units with designated borders).

Both the settlement-related and landscape-related criteria were analyzed in reference to the zones with high densities of sensitive receptors. The settlement-related criteria were defined as settlement areas with the buffer (the distance with the assumed wind farm impact on inhabitants). The landscape-related criteria were defined as the areas important in the country’s nature structure and those with outstanding landscape values (in particular—aesthetic).

#### Settlement-related Criteria

The settlement-related criteria reflect need to protect humans against adverse impacts of wind farms. These were selected with the purpose to ensure a “safe distance” between the installation and places inhabited by people on a permanent basis, as well as those visited such as e.g., recreation areas, schools or hospitals. All wind farm adverse impacts should be encompassed within a safe distance (buffer)—not only sound emissions, but also other impacts affecting human safety, health, as well as mental comfort.

As pointed out earlier, from the perspective of local communities, a certain threshold distance between wind turbines and residential areas should be maintained (Sturge et al. 2014; Van der Horst and Toke 2010). Noise predictions for a given installation are decisive for determination of distance from a residential area. In the majority of the EU’s member states, legislation regarding wind farm annoyance concerns only wind turbine noise (acoustic standards in law regulations), as there is a consensus that excessive noise has an adverse impact on human health (WHO Europe 2011). Increasing populations that live too close to wind farms are perceived as an undesirable side effect of the development of wind energy (Möller 2010). In Poland, the regulation on keeping the minimum distance between wind farms and residential areas (10 times the turbine height) entered into force in 2016 (before, in a planned law regulation, the minimum distance proposed was 3000 m—totally unrealistic under Polish conditions).

Many studies indicate that the perception of any wind turbine impact, including that sound-induced, is associated with wind farm visibility in the landscape. Indeed, turbine visibility enhances experiencing annoyance (Pedersen 2011; Pawlaczyk-Łuszczyńska et al. 2014). A pilot study carried out in Poland by Pawlaczyk-Łuszczyńska et al. (2014), estimated a distance between a wind farm and buildings in the context of human comfort. The authors reported that at the distance ranging from 800–1200 m, 23.3 % of respondents perceive noise as irritating if they were outside the buildings, whereas if indoors—14 % of respondents. However, for certain activities carried out in the open air (e.g., resting, walking), irritation due to noise significantly decreased with the increasing distance from a wind farm: 24–31 % at 400–800 m distance and 5–7 % at 800–1200 m. The authors concluded that considerable reduction of annoyance at 800–1200 m allows to assume the distance about 1000 m as sufficient for decreasing annoyance due to wind farms (Pawlaczyk-Łuszczyńska et al. 2014). The similar distance was also proposed in other countries (Hall et al. 2013; House of Lords 2010; Watson et al. 2012).

In our analysis, the settlement-related criteria took into account urban areas (within their administrative borders) and rural residential areas (with 1000 m wide buffer zone). The latter include dense and dispersed settlements. We took into account that all significant wind turbine impacts on humans should be encompassed within a 1000 m wide zone. So as to illustrate the effects of the arbitral adoption of the distance criterion, there also were carried out additional analyses with regard to 2000 m wide buffer zone.

#### Landscape-related Criteria

First, self-evident landscape-related criterion on constraining wind farm siting are the areas protected by legal regulations on nature and landscape. The nature conservation system in Poland consists of 4 area-based protection forms ranked differently in terms of conservation restrictions,^1^ as well as comprises Natura 2000 sites (a network of nature protection areas in the territory of the European Union). National parks and nature reserves are the highest-rank forms of nature protection (totally excluded from any economic activities). These protected areas represent a very strong, decisive criterion. In the case of the remaining two forms of nature conservation, i.e., landscape parks and protected landscape areas (less restrictive), the possibility of siting wind turbines is determined through SEAs and EIAs (on a mandatory basis). Likewise, wind farm siting within the Natura 2000 network is decided through the same procedures.

In the analyses carried out in the present study, all the aforementioned protected areas were classified as limited for wind farm siting. These were considered as the key areas in view of nature protection, as they comprise unique and irreplaceable biological diversity (Huber et al. 2010). Poland’s system of area-based nature protection forms is of comprehensive nature: it serves not only conservation purposes, but also the protection of cultural resources and recreational values. All the three aspects of sustainable development (ecological, economic, and socio-cultural) justify the exclusion of protected areas from wind farm siting.

Taking into account the connectivity of Poland’s nature conservation system, a network of ecological corridors established in the country was also classified as one of the important constraints on wind farm siting. Local disturbances within ecological corridors can result in considerable changes in ecological network functioning at regional and higher than regional levels (Chmielewski 2013; Huber et al. 2010; Richling and Solon 2011). Poland’s network of ecological corridors (comprising forest complexes, wetlands, and surface waters) was designated under Article 10 of Council Directive 92/43/EEC of 21 May 1992 on the conservation of natural habitats and of wild fauna and flora. In accordance with Poland’s law, wind farm siting in any forest area is banned, yet, the issue is controversial. In fact, some authors believe that forest areas are the most suitable for wind farm siting, which is justified by the minimization of adverse impacts of wind installations on the visual environment (Cowell 2010). In the present study, forest complexes were excluded from wind farm siting due to recognition of their natural and social functions.

Landscape values (aesthetic qualities) are included into consistent and complex Poland’s nature conservation system, as well. The areas with outstanding and high landscape values were classified as the constraint for wind farms. For the purpose of this study, there was performed environmental evaluation (valorization) of Poland. The country’s natural landscapes were valorized at a regional level, taking into account spatial differentiation of habitats, the associated with the mosaic of topographical relief and waters. The following features were evaluated: landscape morphology, surface waters (as: rivers, lakes and water reservoirs) and vegetal cover (in particular: forests). The landscape valorization was performed in accordance with the 4-degree scale: the areas with (1) outstanding, (2) high, (3) medium, and (4) low landscape values. In Poland, the overwhelming majority of the areas with outstanding and high landscape values are situated within nature conservation areas. It should be emphasized again—within many of Poland’s protected areas, the object of protection is the landscape—not only that natural, but also—cultural Unquestionably, the majority of Poland’s highly valuable cultural landscapes (e.g., architectural monuments) is related to residential areas (in the present study excluded from wind farm siting in view of the protection of human life conditions).

### Set of Criteria

The proposed set of criteria is original, however, it reflects Poland’s spatial and ecological policies and regulations (Table 2). Some of the criteria are based on national law and unambiguously decide upon the exclusion of a given area from the development of wind energy. Other criteria in the set refer just to limitations (of different types). The latter have been used to various degrees in several different management plans (at a regional level) prepared with regard to wind farm situation (Borzyszkowski and Cichocki 2010; Degorski 2012; Kubicz et al. 2003; Michalczuk 2011; Olech and Juchnowska 2006; Zathey 2010), as well as in the studies by Baban and Parry (2001), Fiutowska and Dąbrowski (2013), Kistowski (2012), Sliz-Szkliniarz and Vogt (2011), Synowiec and Luc (2013).

**Table 2:** Selected criteria on wind farm siting

Criteria		Comments
*Settlement-related criteria*		
Areas of cities within their administrative boundaries		Legal criterion. Wind farm siting is prohibited
Residential areas with a buffer zone (1000 and 2000 m)		Legal criterion. Wind farm siting is prohibited. Buffers—as classified in the study
*Landscape-related criteria*		
Areas protected as part of the National System of Protected Areas	National parks and nature reserves	Legal criterion. Wind farm siting is prohibited
	Landscape parks and protected landscape areas	Legal criterion. Wind farm siting is permitted if the environmental impact assessment demonstrates that its impact would be tolerable
Areas protected as part of the European Ecological Network Natura 2000		Legal criterion. Wind farm siting is permitted if the environmental impact assessment demonstrates that its impact would be tolerable
Forests		Legal criterion. Wind farm siting is prohibited
Surface waters and wetlands		Legal criterion. Wind farm siting is prohibited
Ecological corridors established pursuant to Directive92/43/EEC		As classified in the study.. Corridor range includes forest complexes, wetlands and surface waters
Areas with outstanding and high landscape values		As classified in the study

## Methods

### Study Location

**Fig. 4:**
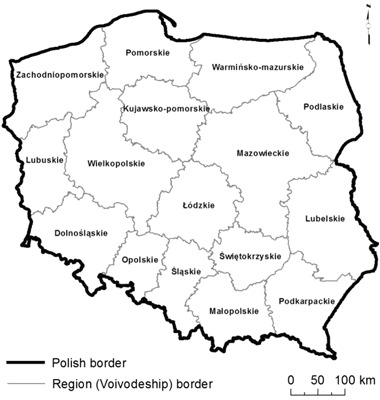
Poland’s division into regions (Voivodships)

The section below describes the study area, with reference to the selected features of the regions (Voivodships, i.e., the areas administered by the Governors). The analysis concerned the territory of Poland, divided into 16 Voivodships (NUTS-3), exclusive of the Baltic Sea (Fig. 4). Poland’s regions are dissimilar in terms of landscape and socioeconomic conditions. The selected features (regional differentiation indicators) are presented in Table 3.

**Table 3:** Natural and socioeconomic differentiation of Voivodships in Poland

Voivodships (regions)	Shares of NSPNA^a^ areas and Natura 2000 sites [%]	Forest cover [%]	Average population density [person /km^2^]	Settlement network density^b^ [km^2^/settlement unit]	Degree of rural settlement dispersal^c^ [%]	GDP per capita (2014) [%, PL = 100 %]
Poland	43.14	29.4	122	4.1	2.8	100
Dolnośląskie	35.02	29.7	144	5.2	2.5	113.1
Kujawsko-Pomorskie	32.45	23.4	115	3.6	2.0	81.3
Lubelskie	36.34	23.1	86	4.7	3.2	70.3
Lubuskie	46.41	49.2	72	4.9	2.6	83.1
Łódzkie	32.78	21.3	140	2.8	3.1	93.2
Małopolskie	59.48	28.6	216	5.3	4.4	88.1
Mazowieckie	39.82	23.0	146	3.1	3.1	159.2
Opolskie	31.53	26.6	110	5.5	2.2	80.8
Podkarpackie	53.47	37.9	118	6.3	4.3	70.0
Podlaskie	43.71	30.7	59	3.6	2.6	71.7
Pomorskie	48.38	36.3	121	3.7	2.3	97.9
Śląskie	33.15	31.9	377	6.1	3.0	105.8
Świętokrzyskie	65.27	28.9	109	3.3	3.6	75.0
Warmińsko-Mazurskie	56.19	33.7	59	3.8	1.9	71.7
Wielkopolskie	36.82	26.8	114	3.9	2.2	106.3
Zachodnio-pomorskie	47.16	38.4	74	4.3	1.7	84.3

*Source*: Central Statistical Office of Poland 2014

^a^ National System of Protected Natural Areas

^b^ Settlement network density measured by the size of the area per settlement unit (excluding wooded and water areas)

^c^ Settlement network dispersal is defined by the share of built up farmland in the total surface area of farmland (according to the geodetic classification). This indicator should be treated as an indicative one, since it also covers built up farmland (farmhouses and related outbuildings) situated within a compact settlement system. The indicator defines which part of the agricultural space is settled

**Fig. 5:**
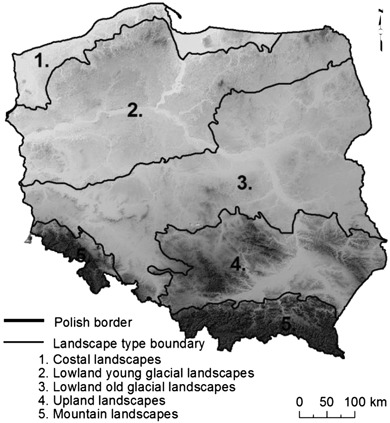
Landscape pattern in Poland

Poland comprises band, latitudinal pattern of landscapes, matching different morphogenesis of relief—from mountainous landscapes in the south, through uplands and lowland landscape belts (originating from the Vistulian and older glaciations), to the coast of the Baltic Sea in the north of the country (Fig. 5). This pattern also shapes Poland’s climate, including wind conditions.

Poland’s physiography is reflected in regional dissimilarities, also those with regard to natural and landscape values. The most valuable landscapes are related to the mountains as well as lake and coastal areas. All these encompass biodiversity abundance, which is reflected in the fact, that there have been designated numerous areas under legal protection. In total, within 6 out of 16 Poland’s Voivodships, comprising mountains, lakes or coastal areas (Małopolskie, Podkarpackie, Świętokrzyskie, Zachodniopomorskie, Pomorskie and Warmińsko-Mazurskie), the protected areas constitute almost 45 % of all the protected areas designated in the country (Table 3). Spatial distribution of natural and landscape values greatly coincides with forest cover distribution. Likewise in the case of protected areas, forest cover proportions are the highest in the regions with mountainous landscapes (Voivodships: Dolnośląskie, Śląskie and Podkarpackie) as well as in lake and coastal landscapes (Voivodships: Zachodniopomorskie, Pomorskie, Warmińsko-Mazurskie and Lubuskie) (Table 3).

Considerable differentiation between the regions is also reflected by social and economic aspects. In Poland, there stand out the Voivodships comprising agglomerations and urban centers, including the capital city of Warsaw (Mazowieckie), Wrocław (Dolnośląskie), Poznań (Wielkopolskie) and the Silesian agglomeration (see Table 2 with GDP per capita). Five regions classified as East Poland (Warmińsko-Mazurskie, Podlaskie, Lubelskie, Podkarpackie and Świętokrzyskie Voivodships) show the lowest level of economic development and represent the least populated regions. Furthermore, socioeconomic differences between the regions result from Poland’s difficult and complex history, which, among others, affected settlement structure, density, and dispersal (Table 3).

High density of the settlement network is observed in the regions in central Poland, contrary to those situated in southern Poland. In the latter, the settlement patterns show considerable dispersal, resulting from quite sizeable settlement units (often more than 1000 residents/unit). Another case is that of the regions in western and northern Poland. Above all, the latter show a relatively low level of settlement dispersal.

It is worth noting that a dense settlement network not always concur with high population density (e.g.,. Śląskie Voivodship, Table 3). This results from the specific settlement pattern, i.e., high population density in large settlement units.

The differentiation of the regions in terms of natural, social, and settlement-related features as described above, unquestionably affects regional differentiation in terms of spatial constraints on wind farm siting as well as wind energy development. Therefore, the criteria related to these aspects were used in the analyses carried out.

### Data and Tools

Relevant data was gathered appropriately to the criteria selected. Reference data was obtained from the national databases (of the Centre of Geodetic and Cartographic Documentation—CODGiK and the General Directorate for Environmental Protection—GDOŚ): the General Geographic Objects Database—BDOO and the Central Register of Nature Conservation Forms. The vector data obtained was presented in PL-1992 plane rectangular coordinate system, according to the National Spatial Reference System (GRS-80 ellipsoid in the Gauss-Krüger projection). BDOO database was founded on the Topographic Objects Database, which was established at an accuracy corresponding to the scale of 1:10 000 (BDOT10k).

The class: Buildings comprises built up areas with an area at least 25,0000 m^2^. Several types of this class objects were distinguished, and this allowed for excluding industrial and storage buildings (insensitive to wind farm impacts) from the analyses. The selected vector data representing the areas designated according to the criteria (Table 2), used in the analysis, allowed to determine the areas with limited possibilities of wind farm siting. With regard to residential areas (buildings), the distance-based criterion was applied: (1000 and 2000 m wide buffer zones).

ArcGIS software, version 10.0, was used in the analyses.. ArcGIS statistical tools were used in calculations of the following indicators for the criteria: (1) the proportion [%] of nature and landscape valuable areas in the total region area (for landscape-related criteria), and (2) the proportion [%] of residential areas (including 1000 or 2000 m wide buffer zones) in the total region area (for the settlement-related criteria). The comparison of the indicators enables evaluating the importance of a given group of criteria in constraining wind farm siting within a given region. At the same time, there was calculated the conclusive indicator, i.e., the proportion [%] of areas excluded from wind farm siting in each region. This indicator directly shows the differences between the regions with respect to the constraints on wind energy development.

## Results

The analysis and spatial identification of sensitive receptors of wind farm impacts allowed to determine regionally differentiated constraints on wind energy development. The results obtained using the landscape-related and settlement-related criteria show that Poland’s territory is substantially limited for wind energy development. Table 4 presents the determined constraints and their regional differentiation.

**Table 4:** Regionally differentiated constraints on wind energy development in Poland

Voivodships (regions)	Share of areas excluded from wind farm siting [%]			Constraints on wind energy development (conclusive indicator) [%]	
	Landscape-related criteria	Settlement-related criteria			
		1000 m buffer zone	2000 m buffer zone	1000 m buffer zone	2000 m buffer zone
Poland	74.8	59.7	86.8	93.9	99.1
Dolnośląskie	73.6	66.7	92.0	94.3	99.8
Kujawsko-Pomorskie	62.8	48.2	81.3	83.4	95.7
Lubelskie	75.9	66.5	93.0	95.5	99.6
Lubuskie	92.6	40.5	74.4	98.3	100.0
Łódzkie	40.8	73.4	96.2	89.7	99.0
Małopolskie	87.0	84.7	95.6	99.9	100.0
Mazowieckie	59.4	68.7	93.1	90.6	98.6
Opolskie	53.5	65.7	94.6	88.6	99.7
Podkarpackie	91.6	64.7	84.7	99.2	100.0
Podlaskie	76.2	47.2	78.7	93.3	99.0
Pomorskie	88.9	50.2	81.1	96.0	99.4
Śląskie	59.3	85.5	97.5	98.4	100.0
Świętokrzyskie	92.5	80.4	97.4	99.6	100.0
Warmińsko-Mazurskie	94.9	36.8	73.1	97.3	99.1
Wielkopolskie	67.0	57.0	87.8	90.0	98.7
Zachodniopomorskie	87.0	41.3	76.3	94.8	99.2

In the regions with lower nature and landscape values, the settlement-related criteria are of great importance (decisive), particularly in the case of 2000 m wide zone around a residential area. In the Śląskie Voivodship, even though the settlement network is relatively sparse (Table 2), the settlement-related criteria showed the strongest effects. This region shows the largest share of urbanized and built up area (13.9, and 12.2 % when industrial areas are excluded)—compared to the country’s average (6.9, and 6.5 % when industrial areas are excluded). Also, in this region, the average size of a single settlement unit is the largest (3300 residents/unit compared to the country’s average—778 residents/unit). Likewise, in the Opolskie Voivodship, stronger effects of the settlement-related criteria were observed when compared to those related to the landscape-related criteria. At the same time, Mazowieckie Voivodship, as well as in the Łódzkie Voivodship, the importance of the settlement-related criteria results from the dense settlement network. In the case of the Małopolskie Voivodship, considerable settlement dispersal (Table 2) prioritize the settlement-related criteria. Hence, the importance of the settlement-related criteria can be associated either with the settlement proportion in the region area or the settlement unit size (the number of residents) or the settlement network density or settlement dispersal. The arbitrarily adopted distance between wind farms and residential areas is a very strong constraint on wind farm siting. This is particularly evident, when there are compared the effects of 1000 and 2000 m buffer zones.

When 2000 m wide buffer zone is assumed, the constraints on wind energy development greatly increase. At this distance, the conclusive indicator value increases by several percent on a country scale and it can achieve 100 % in some regions (Table 4).

**Fig. 6:**
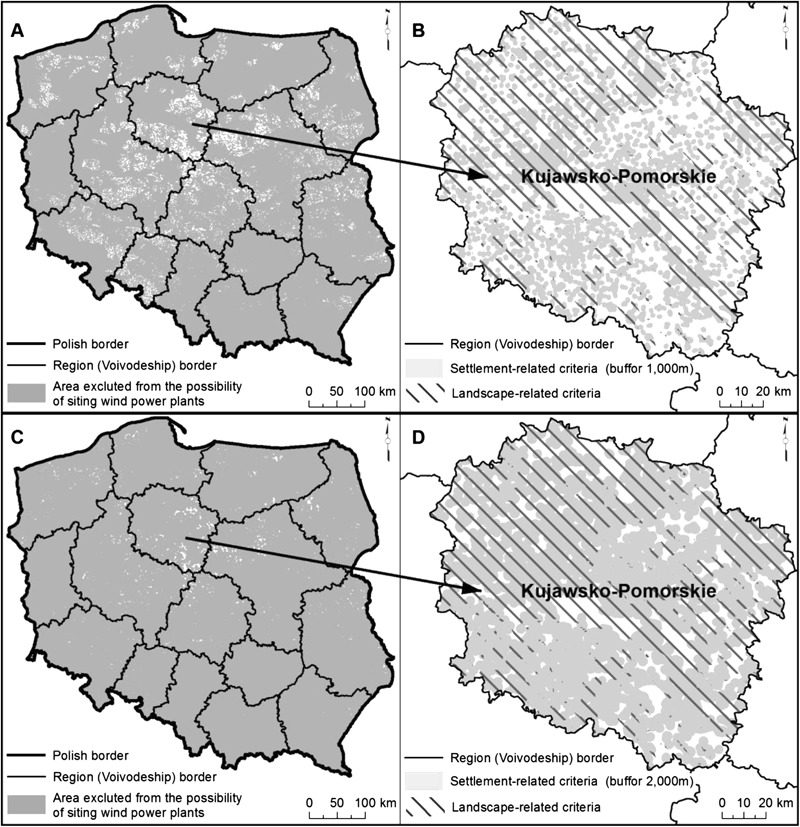
Constraints on wind energy development—at a distance of 1000 m from buildings **a** in Poland and **b** in sample region—at a distance of 2000 m from buildings **c** in Poland and **d** in sample region

The cartographic display of the constraints (Fig. 6) shows that the distribution of the areas excluded from wind farm siting, clearly reflects Poland’s landscape patterns as well as situation of nature conservation areas (described in Section 3). Therefore, evident constraints on wind farm development were determined in the lake landscapes in northern Poland as well as those mountainous in south-eastern and south-western Poland (Fig. 5).

The areas with the hilly relief and rich hydrographic network, i.e., lake districts in northern Poland, are characteristic of the mosaic structure that favors biodiversity and enhanced sensitivity to human-induced impacts. Poland’s lake districts comprise habitats of numerous bird species (very sensitive receptors of wind farm impacts), protected at the national (Act on Nature Conservation 2004) and international levels (Bonn Convention 1979; Directive 2009/147/EC; Ramsar Convention 1971). Moreover, the boundary of the range of many migratory bird species runs across northern Poland (Sikora 2007).

Most of the mountainous landscapes in the Carpathians and the Sudety Mts. are under legal protection (Table 4). These are often covered with forests, whereas diversified relief of farmland areas forms the mosaic landscape structure. In the Carpathian region, the mosaic is enhanced by fragmented land ownership (numerous field borders, differentiated crops). For self-evident reasons, the mosaic landscape structure imposes serious constraints on wind farm siting (just as the fragmented settlement network does). When compared with other Poland’s regions, the greatest constraints on wind farm siting were determined in mountainous landscapes.

A wide land strip running across central Poland (excluding forested areas in the west) encompass the majority of relatively unconstrained areas as regards wind farm siting. The regions here comprise lowland landscapes with lower values. Most often, these are deforested areas of denuded moraine plateaus, and sometimes—sandur areas with less diversified relief when compared with the areas in northern (lake and coastal landscapes) or southern (mountainous landscapes) parts of Poland.

## Discussion

The present study showed that the use of specific criteria (settlement-related and landscape-related) in evaluation of possibilities of wind farm siting substantially reduces availability of space for wind energy development. In the discussion on the results obtained, the following three aspects should be borne in mind: the effect of the level of analyses (1) and the effect of the criteria used (2) on the results obtained. The 3rd issue concerns shaping energy policy as well as its implementation. The three aspects are broader discussed below.

### Levels of Analysis

The identification of the receptors sensitive to wind farm impacts requires distinction between the regional and local level of analyses, followed by the selection of relevant criteria. The analysis level determines the possibilities and purposefulness of identifying the sensitive receptors, as well as a manner to do so. The sensitive receptors are the same at the regional and local levels, i.e., man and the landscape. However, they can be identified in different ways, depending on the adopted level of analyses. At a regional level, the sensitive receptors are identified by determination of the zones with high density of sensitive receptors. At a local level, the identification concerns one wind farm (or individual installation), and the adverse impacts of an investment project on the sensitive receptors are evaluated. Therefore, certain receptors can be identified at a local level only. Above all, this concerns the structure and functioning of environment elements, such as e.g., nature. Natural quality of a given area (mainly species diversity) is strongly related to the landscape mosaic that cannot be fully recognized in regional level analyses.

Likewise, landscape aesthetic values should be definitely evaluated at a local level. The analysis of wind farm visual impact is possible, only when the local cultural and natural landscapes are taken into account. Visual impact assessments should be based on the identification of the perception points (active exposure), scenic axes and foreground scenic quality (passive exposure), as well as the relationships between all the elements of the composition.

It is evident, that the available space for wind farm siting determined with the use of the analysis at a regional level is substantially reduced in comparison with the result of the analysis at a local level. This was experienced by the authors of the present study, when they investigated the possibilities of wind energy development in the Podkarpackie Voivodship (Borzyszkowski and Cichocki 2010). The local criteria were applied to the areas determined at a regional level, and therefore, a substantial decrease of the area available for wind farm siting was observed.

Accordingly, there can be expected that truly existing constraints on wind farm siting would be revealed as a result of more detailed analyses, based on better recognition of environmental conditions at a local level.

### Selection of Criteria

In the present study, there were adopted specific criteria with reference to environment sensitivity to adverse wind farm impact. However, wind farm siting depends upon numerous factors, including technical considerations and wind conditions. If Poland’s regions, were evaluated with regard to unfavorable wind conditions, the available space for wind energy development would be decreased. For the most part, this concerns Poland’s southern regions, with relatively less favorable wind conditions. In Poland’s northern regions, wind energy development will not be affected, because of high wind energy capacity (Lorenc 2005; Michalczuk 2011; Sliz-Szkliniarz and Vogt 2011).

Obviously, the set of adopted criteria decided upon the results obtained. However, Sliz-Szkliniarz and Vogt (2011) used a broader spectrum of criteria in the Kujawsko- Pomorskie Voivodeship, which also included planned protected areas, technical criteria as well as wind energy capacity. Even so, the area available for wind energy development in the region studied was greater when compared to that in our study. The reason behind this is that Sliz-Szkliniarz and Vogt (2011) used less detailed data when compared to that used in the present study.

Difference between the weights of criteria are of great importance (Baban and Parry 2001). In the present study, the weights of the criteria used were differentiated, but evaluations were not the purpose of the study. Less strictly protected areas (e.g., landscape parks, areas of protected landscape, Natura 2000 sites, especially valuable landscape areas, ecological corridors) restrict wind farm siting to some extent, nonetheless, they do not entirely exclude a possibility to establish a wind farm. On the other hand, however, one could expect that wind energy development within these areas will be considerably limited, among others, due to the lack of approval by local communities.

It seems that, the precautionary principle is the issue of key importance in the adoption of the criteria at a regional level. According to Sliz-Szkliniarz and Vogt (2011): “On a regional scale, the precautionary principle should be followed to avoid any detrimental impact on sensitive areas, since the environmental impact assessment is performed on local level.”

### Energy Policy

The results presented demonstrate the great importance of space availability as the factor affecting wind energy development in Poland. The available space is limited to a substantial extent mainly due to settlement patterns in the country, i.e., strong settlement dispersal. This factor is also important in decision making processes on permissible distance between wind turbines and settlement areas.

As already mentioned, in Poland, there has been recently established the legal threshold (minimum) distance between a residential area and a wind farm, as a result of the failure of the system of spatial planning and environmental impact assessments in terms of meeting the needs of society and nature protection. Michalczuk (2011) refer to an example of ornithologists, who have been skeptical about wind farms from the start of wind energy development in Poland. The “Guidelines on the assessments of the impacts of wind power plants on birds” (Chylarecki and Paslawska 2008)—elaborated and pushed through by Poland’s ornithologists—have been unquestionably implemented ever since, even though the recommended practice is time-consuming and the investors have to meet all the costs of its implementation. The aforesaid assessment of the impacts allows to appropriately situate wind farms, so as to avoid adverse effects on birds and bats (Kepel et al. 2011). The recommendations on other receptors (e.g., landscape) have not been yet elaborated in Poland, and neither developers nor environmental protection bodies have undertaken sufficient activities toward the protection of Poland’s landscape. Therefore, it seems that the stage of investment planning can plays an important role in decisions on wind farm siting. First of all, the system of spatial planning accompanied by SEAs and EIAs should be reliable and transparent for participating local communities (Cowell 2010; Hull 1995; Sliz-Szkliniarz and Vogt 2011; Van der Horst and Toke 2010). The development of wind energy in the harmony with the environment would be possible, if there was made full use of the tools provided by the system of environmental assessments (Hull 1995). The latter should be consistent and utilized at all the levels of creation and implementation of energy policy. This would allow to prevent wind farm siting within the most valuable natural areas with dispersed settlement patterns, as shown by the results of the present study.

### Limitations of the Study

The limitations of the study are related to the issues described above: the level of analysis (here: regional), the level of data precision (here: regional data) and the selection of criteria (here: settlement and landscape-related). Most probably, more precise results would be obtained based on detailed data and considering additional criteria. The differences of the weights of the adopted criteria, as done in other studies at a regional level (Cowell 2010; Kistowski 2012; Sliz-Szkliniarz and Vogt 2011), could also enable better recognition of spatial differentiation with regard to the constraints on wind farm siting.

## Conclusion

The results of the analyses performed demonstrate the importance of the settlement patterns as well as natural and landscape values in decisions on wind farm siting. In view of energy policy, the results of the analyses indicate that in Poland, there exist serious constraints on wind farms, but this does not mean withdrawal from wind energy development. At the same time, in view of local communities’ attitudes, we cannot expect easing the requirements on wind farm siting (in particular: decreasing the distance between wind turbines and residential buildings). The environmental standards adopted by Poland’s law are also increasingly rigorous. Under these circumstances, the success of wind energy implementation in Poland depends on taking into account the above restrictions as well the standards for wind farm siting, including the wind farm size, when determining farm localization. The process of determination must be carried out in a credible manner to meet the expectations and needs of local communities. There should be emphasized, however, that the standards (and especially, the distance from residential areas) should not depend only on noise propagation (that will be gradually decreased as technology advances). As stressed by numerous authors, wind farm visual impact has recently become increasingly important. Wind farm visual impact is impossible to eliminate. Taking into consideration all the above, we cannot conclude that the restrictions on wind farm siting would be easy to pass over, especially when the priority is given to the protection of human wellbeing and landscape values, and not to wind farm investment projects.

## Footnotes

1 National parks, nature reserves, landscape parks and protected landscape areas (Act of 16 April 2004 on Nature Conservation, consolidated text in Official Journal of the Laws of 2013, Item 627).

## References

[CR1] Act of 16 April on Nature Conservation (2004) Consolidated text in Official Journal of the Laws of 2013, Item 627

[CR2] Agterbosch S, Meertens RM, Vermuelen WJV (2009). The relative importance of social and institutional conditions in the planning of wind power projects. Renew Sust Energ Rev.

[CR3] Baban SMJ, Parry T (2001). Developing and applying a GIS-assisted approach to locating wind farms in the UK. Renew Energ.

[CR4] Baxter J, Morzaria R, Hirsch R (2013). A case-control study of support/opposition to wind turbines: perceptions of health risk, economic benefits, and community conflict. Energ Policy.

[CR5] Bidwell D (2013). The role of values in public beliefs and attitudes towards commercial wind energy. Energ Policy.

[CR6] Borzyszkowski J, Cichocki Z (2010). A study on the landscape, natural, cultural and tourism-related factors affecting wind energy development in Podkarpackie Voivodship (in Polish).

[CR7] Bradley MP, Smith ER (2004). Using science to assess environmental vulnerabilities. Environ Monit Assess.

[CR57] Breukers S, Wolsink M (2007). Wind energy policies in the Netherlands: Institutional capacity-building for ecological modernisation. Environmental Politics.

[CR8] Central Statistical Office of Poland (2014) http://stat.gov.pl/en Accessed 16 Aug 2014

[CR9] Chmielewski TJ (2013). Landscape systems. Structure – functioning – planning (in Polish).

[CR10] Chylarecki P, Paslawska A (2008) Guidelines on the assessments of the impacts of wind power plants on birds (in Polish). PSEW. Szczecin

[CR11] Convention on the Conservation of Migratory Species of Wild Animals (1979) Bonn http://www.cms.int/ Accessed 12 Oct 2014

[CR12] Convention on Wetlands. (1971). Ramsar http://www.ramsar.org/ Accessed 12 Oct 2014

[CR14] Cowell R (2010). Wind power, landscape and strategic, spatial planning – The construction of ‘acceptable locations’ in Wales. Land Use Policy.

[CR15] Degorski M (Ed.) (2012) Wind energy in the context of the protection of the natural and cultural landscapes in Kujawsko-Pomorskie Voivodship (in Polish). Institute of Geography and Spatial Organization of the Polish Academy of Sciences in Warsaw. Warsaw

[CR16] Directive (2009) /147/EC of the European Parliament and of the Council of 30 November 2009 on the conservation of wild birds http://eur-lex.europa.eu/ Accessed 12 Oct 2014

[CR17] Ek K, Persson L, Johansson M, Waldo A (2013). Location of Swedish wind power – Random or not? A quantitative analysis of differences in installed wind power capacity across Swedish municipalities. Energ Policy.

[CR18] Eltham DC, Harrison GP, Allen SJ (2008). Change in public attitudes towards a Cornish wind farm: Implications for planning. Energ Policy.

[CR19] Energy Policy of Poland until 2030 (2009) Resolution no. 202 of the Council of Ministers of 10 November 2009 http://mg.gov.pl Accessed 14 Jan 2014

[CR20] Energy Regulatory Office of Poland (2014) http://ure.gov.pl Accessed 4 Feb 2014

[CR21] Fiutowska G, Dąbrowski Ł (2013). The wind energy development in the light of planning and landscape terms on examples of Puck and Gniewino communities and the city of Gdynia (in Polish). Problems Landscape Ecol.

[CR22] Groth TM, Vogt Ch (2014). Residents’ perceptions of wind turbines: an analysis of two townships in Michigan. Energ Policy.

[CR23] Hall N, Ashworth P, Devine-Wright P (2013). Societal acceptance of wind farms: analysis of four common themes across Australian case studies. Energ Policy.

[CR24] House of Lords (2010) Wind Turbines (Minimum Distances from Residential Premises) Bill [HL], July 26. UK Parliament, London

[CR25] Huber PR, Greco SE, Thorne JH (2010). Spatial scale effects on conservation network design: tradeoffs and omissions in regional versus local scale planning. Landscape Ecol.

[CR26] Hull A (1995). Local strategies for renewable energy policy approaches in England and Wales. Land Use Policy.

[CR27] Kepel A, Ciechanowski M, Jaros R (2011). Guidelines on the assessments of the impacts of wind power plants on bats - project (in Polish).

[CR28] Kistowski M (2012). A methodological proposal for regional-scale assessment of environmental conditions in relation to the establishment of wind farms (in Polish). Polish. Geogr Rev.

[CR29] Kokologosa D, Tsitouraa I, Kouloumpisb V, Tsoutsosa T (2014). Visual impact assessment method for wind parks: a case study in Crete. Land Use Policy.

[CR30] Kostrowicki AS (1992) The “man – environment” system in the light of evaluation theory (in Polish). Geographical Studies 156. Institute of Geography and Spatial Organization of the Polish Academy of Sciences in Warsaw Wrocław-Warsaw-Cracow

[CR31] Kubicz G, Wojcieszyk H, Wojcieszyk K, Musiał R, Brokos B (2003). A study on the possibilities of wind energy development in Pomorskie Voivodship (in Polish).

[CR32] Kunz TH, Arnett EB, Cooper BM, Erickson WP, Larkin RP, Mabee T, Morrison ML, Strickland MD, Szewczak JM (2007). Assessing impacts of wind-energy development on nocturnally active birds and bats: a guidance document. J Wildlife Manage.

[CR33] Lorenc H (2005). An atlas of Poland’s climate (in Polish).

[CR34] Michalczuk W (2011). The spatial aspects of wind energy siting in Lubelskie Voivodship (in Polish).

[CR35] Möller B (2010). Spatial analyses of emerging and fading wind energy landscapes in Denmark. Land Use Policy.

[CR36] Olech S, Juchnowska U (2006). T. he natural and spatial aspects of wind energy siting in Warmińsko-Mazurskie Voivodship (in Polish).

[CR37] Pavlickova K, Vyskupova M (2015). A method proposal for cumulative environmental impact assessment based on the landscape vulnerability evaluation. Environ Impact Asses.

[CR38] Pawlaczyk-Łuszczyńska M, Dudarewicz A, Zaborowski K, Zamojska-Daniszewska M, Waszkowska M (2014). Evaluation of annoyance from the wind turbine noise: a Pilot Study. Int J Occup Med Environ Health.

[CR39] Pearce-Higgins JW, Leigh S, Douse A, Langston RHW (2012). Greater impacts of wind farms on bird populations during construction than subsequent operation: results of a multi-site and multi-species analysis. J Appl Ecol.

[CR40] Pedersen E (2011). Health aspects associated with wind turbine noise – Results from three field studies. Noise Control Eng J.

[CR41] Pepermans Y, Loots I (2013). Wind farm struggles in Flanders fields: a sociological perspective. Energ Policy.

[CR42] Richling A, Solon J (2011). Landscape ecology (in Polish).

[CR43] Rodrigues L, Bach L, Doubourg-Savage MJ, Karapandza B, Kovac D, Kervyn T, Dekker J, Kepel A, Bach P, Collins J, Harbusch C, Park K, Micevski B, Minderman J (2015) Guidelines for consideration of bats in wind farm projects. Revision 2014. EUROBATS publication Series No. 6. Bonn

[CR44] Schuster E, Bulling L, Köppel J (2015). Consolidating the state of knowledge: a synoptical review of wind energy’s wildlife effects. Environ Manage.

[CR45] Sikora A (2007). An atlas of the distribution of Poland’s breeding birds in 1985-2004 (in Polish).

[CR46] Sliz-Szkliniarz B, Vogt J (2011). GIS-based approach for the evaluation of wind energy potential: a case study for the Kujawsko–Pomorskie Voivodeship. Renew Sustainable Energy Rev.

[CR47] Smart DE, Stojanovic TA, Warren CR (2014). Is EIA part of the wind power planning problem?. Environ Impact Asses.

[CR48] Sturge D, While A, Howell R (2014). Engineering and energy yield: the missing dimension of wind turbine assessment. Energ Policy.

[CR49] Synowiec W, Luc M (2013). A multicriterial evaluation of land suitability for wind energy development, as exemplified by Poland’s gmina of Rymanów (in Polish).. Pol Geogr Rev.

[CR50] Toro J, Duarte O, Requena I, Zamorano M (2012). Determining vulnerability importance in environmental impact assessment. The case of Colombia. Environ Impact Asses.

[CR51] Van der Horst D, Toke D (2010). Exploring the landscape of wind farm developments; local area characteristics and planning process outcomes in rural England. Land Use Policy.

[CR52] WHO Europe (2011). Burden of disease from environmental noise. Quantification of healthy life years lost in Europe.

[CR53] Wolsink M (2007). Planning of renewable schemes: deliberative and fair decision-making on landscape issues instead of reproachful accusations of non-cooperation. Energ Policy.

[CR54] Wolsink M (2010). Near-shore wind power – Protected seascapes, environmentalists’ attitudes, and the technocratic planning perspective. Land Use Policy.

[CR55] Watson I, Betts S, Rapaport E (2012). Determining appropriate wind turbine setback distances: perspectives from municipal planners in the Canadian provinces of Nova Scotia, Ontario, and Quebec. Energ Policy.

[CR56] Zathey M (2010). A study on the spatial factors affecting wind energy development in Dolnośląskie Voivodship (in Polish).

